# Rapid plasmid replicon typing by real time PCR melting curve analysis

**DOI:** 10.1186/1471-2180-13-83

**Published:** 2013-04-15

**Authors:** Maikel Boot, Susanne Raadsen, Paul HM Savelkoul, Christina Vandenbroucke-Grauls

**Affiliations:** 1 Medical Microbiology & Infection Control, VU University medical center, De Boelelaan 1117, Postbus 70571007 MB, Amsterdam, The Netherlands

**Keywords:** ESBL, Plasmid, Replicon typing, SYBR-green

## Abstract

**Background:**

Genes encoding Extended Spectrum Beta Lactamases are usually located on transferable plasmids. Each plasmid contains its own replication mechanism. Carattoli *et al*. developed an extended PCR-based replicon typing method to characterize and identify the replicons of the major plasmid incompatibility groups in *Enterobacteriaceae*. Based on this method, we designed a rapid approach with amplicon detection by real-time melting curve analysis. This method appeared to be fast, sensitive, less laborious, less prone to contamination and applicable in a routine laboratory environment.

**Results:**

We successfully integrated the post-PCR analysis of the replicon typing into a closed system, which leads to a 10-fold increase in sensitivity compared to agarose gel visualization. Moreover, the use of crude lysates and SYBR-green saves a considerable amount of hands-on time without decreasing the sensitivity or specificity.

**Conclusions:**

This real-time melting curve replicon typing method appears to be fast, sensitive, less laborious, less prone to contamination and applicable in a routine laboratory environment.

## Background

Extended Spectrum Beta Lactamases (ESBLs) have been reported increasingly often in the last few decades and constitute a serious threat to public health [1,2]. ESBLs are enzymes that give a bacterium the ability to inactivate penicillins, cephalosporins (up to the fourth generation) and monobactams, thereby yielding bacterial resistance to these commonly used antimicrobial agents. Usually, the genes that encode these enzymes are found on plasmids.

Plasmids are extrachromosomal genetic elements that can replicate independently of their host. They consist of double-stranded DNA and carry genes that are non-essential for the host’s growth or survival [3]. Plasmids are found in virtually all bacterial species. These genetic elements can spread vertically from parent to progeny, or horizontally from cell to cell. The size of plasmids can vary from 1 kb up to 400 kb and depends on the amount of genes they carry [4-6]. These genes may include, besides the household genes that regulate the autonomous plasmid replication, virulence genes and antimicrobial resistance genes [7,8]. The presence of antimicrobial resistance genes, and/or virulence genes, and/or toxin-antitoxin genes can result in positive selection of these plasmids in the host and has led to evolution of plasmids over time.

In 1971, Datta and Hedges proposed a method of classification for plasmids [9]. This classification is based on the stability of plasmids during their transmission from host to host. The measure for this stability is ‘compatibility’ and is defined as the ability of two closely related plasmids to stably coexist in the same host cell [10]. If a plasmid cannot co-reside with another plasmid they are said to belong to the same incompatibility group (Inc-group). This incompatibility is due to overlap of the plasmid replication machinery. The replication machinery thus determines the Inc-group of a plasmid. Since Inc-typing is time-consuming, replication machinery typing (replicon typing) is performed more often.

Based on this classification, Carattoli *et al*. designed a PCR-based method to identify the replicons of the major plasmid families that are found in *Enterobacteriaceae*. This method allows discrimination between 18 different plasmids in a multiplex PCR setting with a total of 8 reactions (5 multiplex and 3 simplex reactions). The PCR products are analyzed for size by agarose gel visualization [11]. Recently, Carattoli further updated the typing scheme [12,13].

We adapted Carattoli’s typing method with the aim to speed up the process of analyzing a great number of Enterobacterial isolates for their plasmid content. By adding the fluorescent dye SYBR-green to the PCR-mixture and amplification on a real time PCR platform, we increased the sensitivity of the assay, and simplified the product analysis by substituting the agarose gel visualization by melting curve analysis. The SYBR-green approach eliminates the time-consuming agarose gels and reduces the risk of contamination.

## Results

### Analytical sensitivity and specificity

**Figure 1:**
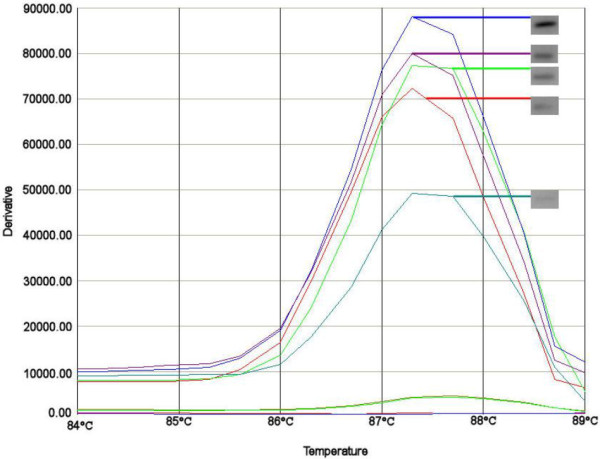
**Melting curves of serial dilutions of the FIIs replicon. **The melting curves intensity differences based on 10^-1 ^to 10^-9 ^dilutions of the FIIs replicon (melting peak at 87.4 average for this experiment). For each melting curve the corresponding agarose band is presented in the grey box. Shown in pairs are the curves that gave a positive result both as melting curve and after visualization on agarose gel (blue = 10^-1^, purple = 10^-2^, green = 10^-3^, red = 10^-4^ and turquoise = 10^-5^). The dilutions from 10^-6^ to 10^-8^ are visible as peaks of 4300 y (10^-6^) to 4117 y (10^-8^). These are not shown as agarose bands because they were not visible on agarose gel.

The analytical sensitivity of the assay was determined with serial concentrations of cloned replicon DNA ranging from 5 ng to 50 fg. For all different clones the PCR showed a clear melting curve position ranging from 82,1°C to 88,9°C (see Table 1). The DNA concentrations varied from 5 ng to 5 fg of vector DNA (estimated number of plasmids for 5 fg: ~1087). Comparison of the melting curve analysis with agarose gel electrophoresis results showed that the sensitivity of the melting curve analysis was tenfold higher than the sensitivity of the agarose method (see Figure 1).

**Table 1:** Average melting temperature of reference amplicons with CV% and SDs

**Replicon name**	**Size of reference plasmid and amplicon (bp)**	**Melting temperature of amplicon (°C)**	**Average TM**	**SD**	**CV%**
A/C	4365	86.3	86.3	0.05	0.06
B/O	4059	85.1	85.1	0.17	0.20
ColE	4087	86.4	86.4	0.20	0.23
ColEtp	4006	84.9	84.9	0.13	0.16
F	4170	84.2	84.2	0.24	0.29
FIA	4362	84.0	84	0.17	0.21
FIB	4602	86.4	86.4	0.07	0.08
FIC	4162	83.6	83.6	0.15	0.18
FIIs	4170	87.7	87.7	0.18	0.20
HI1	4371	83.6	83.6	0.18	0.21
HI2	4544	86.3	86.3	0.11	0.13
I1	4039	83.3	83.3	0.12	0.15
K	4060	85.2	85.2	0.09	0.10
L/M	4685	84.7	84.7	0.08	0.10
N	4459	86.5	86.5	0.17	0.19
P	4434	88.4	88.4	0.15	0.17
R	4151	84.4	84.4	0.18	0.21
T	4650	83.8	83.8	0.19	0.23
U	4743	88.9	88.9	0.09	0.10
W	4142	88.9	88.9	0.09	0.10
X	4276	82.1	82.1	0.22	0.27
Y	4665	86.6	86.6	0.31	0.36

Reference plasmids, sizes and average melting temperatures obtained from at least five crude lysates of the cloned replicon plasmid. The average melting temperatures for replicons from WT strains were identical to those of the cloned replicons.

Specificity of the method was tested by mixing of 3 different plasmids containing cloned replicons in a multiplex PCR reaction (i.e. I1+A/C+ColEtp and FIIs+K+T). The mixture of 5 pg of the three cloned replicons FIIs, K and T and their six corresponding primers lead to distinguishable melting peaks with melting temperatures corresponding to those found in simplex reactions (see Additional file 1).The maximum numbers of different cloned replicons that could be detected in one reaction depended on the temperature of disassociation. All primers sets showed a clear specific melting peak, although at concentrations lower than 5 fg additional aspecific peaks appeared. Because of the overlap of disassociation temperatures we chose to amplify a maximum of 3 different replicons per reaction.

### Replicon typing of plasmids in wild type strains

The same amplification procedure was used on the crude lysates of wild type (WT) strains to evaluate applicability in a routine setting. The wild type plasmids were analyzed in fresh crude bacterial lysates. The lysates were tested in a 10^-1^ to 10^-9^ dilution for each strain. Figure 1 illustrates an example of the results obtained with different concentrations of DNA of an *E. coli* containing replicon FIIs. In a range from 10^-1^ to 10^-5^ the melting curve was clearly visible and the melting temperature was stable. The melting temperature was identical when compared to the melting temperature observed for the cloned replicon. Further dilution of the DNA yielded a negative result. Comparison to agarose gel results showed that the intensity of the bands corresponded with the height of the melting curves (Figure 1).

**Figure 2:**
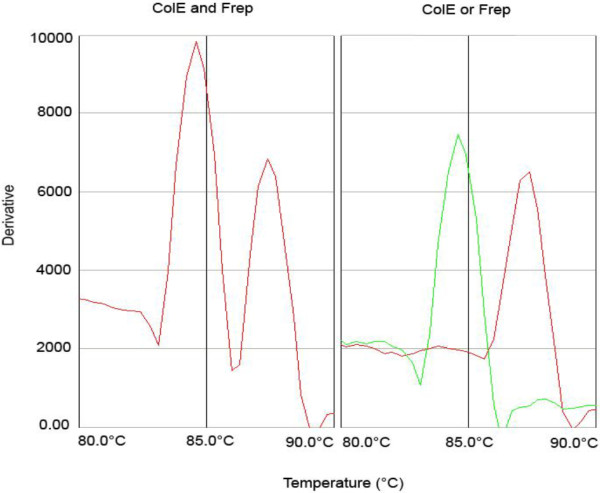
**Detection of multiple WT plasmids shows the same melting curves as corresponding cloned replicon controls. **The left panel shows the melting curve of a WT strain with multiple plasmids. These plasmids were found to be of the ColE and F replicon. In the right panel the same result is obtained from two control strains each bearing either ColE or the F replicon. The melting temperature in the left panel peaks correspond exactly with the right panel peaks at 84.6°C and 86.4°C.

In addition, the presence of more than one plasmid in one strain did not interfere with the accuracy and sensitivity of the melting curve assay (see Figure 2). Figure 2 illustrates that the melting temperature of 84.6°C and 87.4°C from the two positive controls corresponded to the peaks visible in the tested strain.

## Discussion

The emergence of ESBLs has become an imminent threat to public health. This threat is emphasized by the continuous appearance of new β-lactamases. Although not all ESBL-enzymes pose the same threat, some facilitate a wide resistance to first-line antibiotics. To date, more than 900 different β-lactamases have been recognized [14]. Of particular concern is the rapid spread of ESBLs, which is due to the location of the genes that encode them on transferable plasmids in *Enterobacteriaceae*. Identification of these replicons is useful for a better understanding of the epidemiology of the ESBL genes. For replicon identification Carattoli *et al.* developed a multiplex PCR-based replicon typing method [11]. The multiplex approach is very useful, because of the large numbers of different plasmids present in *Enterobacteriaceae*. A drawback is that amplicons have to be identified on agarose gels. We have simplified and quickened the Carattoli PCR by the incorporation of fluorescent dye SYBR-green in a real time PCR. This dye intercalates in the amplicons during the PCR, and is thereby quenched. It is released from the amplicons at specific melting temperature points. Upon release, quenching is abolished and fluorescence can be measured. The use of this dye eliminates the need to detect the amplicons by agarose gel electrophoresis, which means that a time-consuming step is eliminated. Furthermore, since it is not necessary to open the PCR vials for analysis, the risk of contamination by other PCR replicons is decreased. Another advantage of the method we present here is that it is possible to use crude cell lysates in the PCR, with no need to purify plasmid DNA, which is also time and cost saving. The use of crude cell lysates has been described in previous studies and has been shown to provide solid data [15,16].

A third benefit of real time PCR with SYBR-green is its high analytical sensitivity. This is desirable because plasmids can be low-copy-number plasmids and because plasmid numbers vary per bacterial cell and growth phase [17]. In 2011 for instance, Waltner-Toews *et al.* described a wild-type TEM-1-carrying strain, where the plasmid occurred at an average of 3.5 to 4.1 copies per cell [18]. We have shown that we can detect replicons in samples containing as little as 50 fg of DNA (50•10^-15^ g), hence even low-copy-number plasmids can be detected. The dry weight of the average *E. coli* genome of 5 mBp is approximately 5 fg, which means that in theory 10 bacterial cells are needed to be able to detect the replicon [19]. The PCR can be performed with single primer sets or in a multiplex setting. This allows the user to choose between the advantage of high sensitivity or the advantage of multiplexing.

Moreover, 96-wells plates can be used to test 10 strains for up to 8 different plasmid types. Of course, the multiplex setting has its limitations due to an overlap in melting temperatures of some of the replicons. Combinations of replicons should therefore be carefully chosen to allow to discriminate between melting peaks.

Recently, a commercial kit for plasmid typing was introduced (PBRT kit, Diatheva, Fano, Italy). This kit provides the primers and controls needed to run the multiplex PCR, but still requires agarose gels as read out. This makes the kit a less attractive alternative for labs that have access to RT-PCR equipment.

The prevalence of the different plasmid types is variable. For high prevalent plasmids several reference strains are available which can be used as positive controls. For the less prevalent plasmids it is difficult to obtain wild type reference strains. The detection of the replicons in wild type strains will permit to obtain a complete collection of all plasmid types that can serve as positive controls. This is preferable because then the plasmids are studied in their natural plasmid-backbone, which can have specific secondary structures that are lost in cloning vectors like pGEM-T.

## Conclusions

Molecular epidemiologic studies of ESBL genes require ESBL gene characterization, plasmid identification and conjugation experiments, to demonstrate which type of plasmid carries which genes. Our real-time PCR with SYBR green and melting curve analysis simplifies and speeds up the detection and identification of the plasmids, both in wild-type strains and in transconjugants.

## Methods

### Reference strains

Amplified origins of replication of 18 Inc-plasmid types were used as reference templates. The amplicons were cloned in a pGEM-T easy vector in *E. coli DH5α*. A. Carattoli kindly provided these cloned replicons [11]. In addition, three new primer sets were developed by Carattoli to test for ColE, R and U replicons. The same 18 primer sets, used to amplify the 18 Inc-plasmid types were used to detect cloned replicons with the melting curve approach and to identify wild type plasmids. The cloned replicons were isolated with a QIAGEN plasmid kit (Qiagen, Venlo, Netherlands). After isolation, the DNA concentration was calculated with a Nanodrop 2000 (Thermo Fisher Scientific, Wilmington, USA). The cloned replicons were used to determine the analytical sensitivity and specificity of the melting curve approach. A total of 7 reference wild type (WT) strains with known plasmids was used to determine the optimal DNA concentration to detect wild type plasmids. These reference strains can be found in Table 2. The PCR protocol and positive reference strains containing the cloned replicons were kindly provided by A. Carattoli. The strains containing the cloned replicons are under Material Transfer Agreement (MTA) and can be requested through A. Carattoli. Both the reference templates and the WT strains were all grown at 37°C in 5 ml LB broth with 50 μg/ml ampicillin. Plasmids from the WT strains were obtained by suspending single bacterial colonies in 50 μl of distilled H_2_O, heating at 95°C for 5 minutes and centrifugation at 14,000 rpm for 3 minutes. A dilution of this supernatant from the single colony was used for PCR.

**Table 2:** Table of reference strains

**Strain**	**Species**	**Inc Group**	**Paper**
RHH72	*E. coli*	B	Carattoli, A. *et al. *(2005) [11]
R16	*E. coli*	B/O	Carattoli, A. *et al.* (2005) [11]
466444	*E. coli*	FIA, FIB, FIIs, A/C, I1	Gonullu, N. *et al. *(2008) [20]
47731	*E. coli*	FIA, FIB, FIIs, A/C, I1	Gonullu, N. *et al. *(2008) [20]
1185-D	*E. coli*	HI2, FIB, FIIs, Y, N, A/C	Garcia, A. *et al. *(2007) [21]
1185-DT	*E. coli*	HI2	Garcia, A. *et al. *(2007) [21]
1358-TC	*E. coli*	I1	Carattoli, A. *et al. *(2006) [22]
8001	*E. coli*	F, ColE	Overdevest, I. *et al. *(2011)[23]

An overview of the WT strains that were used in this study.

### Real time PCR with SYBR green and melting curve analysis

The PCR mixture consisted of 1 μl of the (diluted) supernatant (100 fg), 400 nM of either single sets or a multiplex set (depending on the experiment) of forward and reverse primers (in a total volume of 11,5 μl distilled H_2_O) and 12,5 μl 2X iQ-SYBR-green supermix (Bio-Rad, Hercules, USA). Amplification was carried out on an Real Time PCR machine (TaqMan 7500, Applied Biosystems, Foster City, USA) with 95°C for 15 min, followed by 32 × 95°C/ 15 s; 65°C/1 min. The subsequent dissociation step consisted of: 95°C/15 s; 60°C/1 min; 95°C/15 s where dissociation was measured stepwise, every 0.5°C. Sequence Detection Software version 1.3.1 (Applied Biosystems) was used to present the resulting melting curves. Agarose gel electrophoresis for control purposes was performed according to the method described by Carattoli in 2005 [11]. Each experiment was performed three times.

## Competing interest

The authors declare that they have no competing interests.

## Authors’ contributions

MB set up the replicon typing with SYBR-Green, participated in determining the sensitivity and specificity of the technique and drafted the manuscript. SR carried out the identification and implementation of the WT strain controls. PHMS and CVG participated in the design of the study and helped to draft the manuscript. CVG conceived the study, and participated in its design and coordination. All authors read and approved the final manuscript.

## Supplementary Material

Additional file 1 **Multiplex reaction of three cloned replicons FIIs, K and T. **Contains a supplementary figure that shows that in multiplex reactions the melting peaks correspond to those found in simplex reactions. Click here for file
