# Supplementary material for: Rapid plasmid replicon typing by real time PCR melting curve analysis
Source: BMC Microbiol. 2013 Apr 15;13:83. doi: 10.1186/1471-2180-13-83 (PMC3639092; doi:10.1186/1471-2180-13-83)
Supplement: Additional file 1 — Multiplex reaction of three cloned replicons FIIs, K and T. Contains a supplementary figure that shows that in multiplex reactions the melting peaks correspond to those found in simplex reactions. [file 1471-2180-13-83-S1.doc]

**Additional file 1**


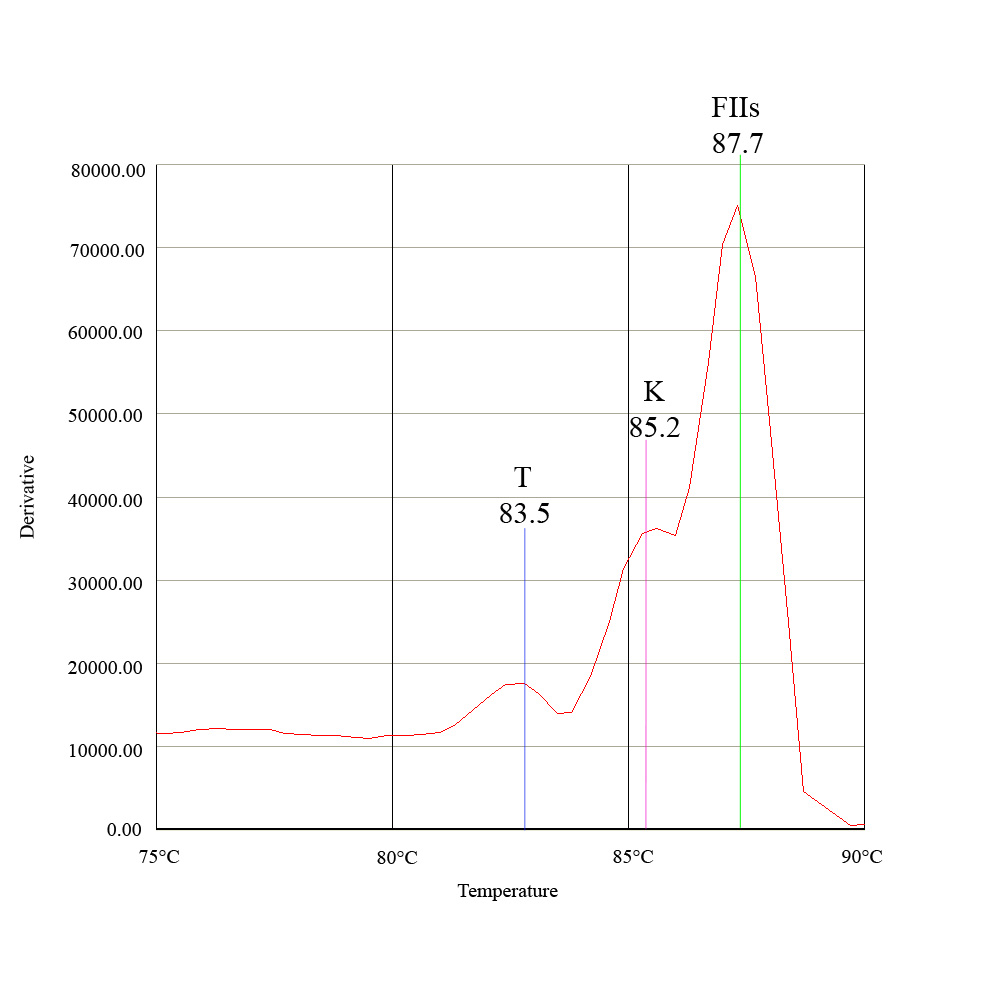


**Multiplex reaction of three cloned replicons FIIs, K and T**

The mixture of three different cloned replicons (5 pg each) in one multiplex shows distinguishable peaks at melting temperatures close to those found in the simplex reactions (Table 1). Shown are replicon T, K and FIIs with the corresponding melting temperatures from this experiment.
